# Benzodiazepine misuse among women: elements for brief intervention

**DOI:** 10.1186/1940-0640-7-S1-A9

**Published:** 2012-10-09

**Authors:** Ana R Noto, Ana R Lins de Souza

**Affiliations:** 1 Department of Psychobiology, Federal University of São Paulo, São Paulo, Brazil

## 

The misuse of benzodiazepines (BZD) among women has raised international concern and interest, but subsidies for prevention are scant. This study aimed at analyzing BZD misuse among women, with an emphasis on the search for subsidies for brief intervention (BI). Thirty-three women aged 18–60 years (median, 46 years) with a history of misuse of BZD in the year comprised the sample. They received a semi-structured interview, the content of which was transcribed and submitted for analysis using NVivo software. Most participants reported a much longer period of BZD use (median of seven years) than is recommended but with medical follow-up and prescription. The reasons reported for use were to reduce anxiety, fight insomnia, or to “escape from problems.” About half of women (n = 14) reported that they intended to continue using BZD despite the possibility of dependence and/or other risks. One-third of the sample reported using strategies to quit and/or reduce use, including praying, practicing relaxation, and receiving psychotherapy. Our results suggest that, although they had medical follow-up, many BZD users in this study had a low perception of the risks of BZD and continued using them for years with no concern for dependence. In such patients, BI might be useful to raise awareness of the BZD risks and the benefits of alternative strategies to cope with anxiety or insomnia.

